# Menstrual hygiene management and associated factors among adolescent school girls in gursum district, Eastern Ethiopia: Institution-based a cross-sectional study

**DOI:** 10.1186/s12905-023-02461-6

**Published:** 2023-06-21

**Authors:** Yohannes Mulugeta Demmu, Gutema Mulatu Shifera, Galana Mamo Ayana, Dechasa Adare, Berhanu Yazew, Yohannes Tefera Damtew, Abraham Geremew

**Affiliations:** 1 School of Environmental Health, College of Health and Medical Science, Haramaya University, Harar, Ethiopia; 2 Department of Epidemiology and Biostatistics, School of Public Health, College of Health and Medical Science, Haramaya University, Harar, Ethiopia

**Keywords:** Menstruation, Menstrual hygiene management, Adolescent girls, Gursum

## Abstract

**Background:**

In 2017 WHO reported that due to a lack of menstrual hygiene management (MHM) facilities, high costs, and ignorance, 2.3 billion girls and women worldwide do not manage their menstruation properly. This leads to the use of other options, such as old clothes or other unhygienic materials, which may make them a risk group for infections and other health consequences. Despite the significant role of appropriate menstrual hygiene practices, it is still a missed opportunity to address the hygienic practice of menstruation among girls in many low-and middle-income countries, including Ethiopia.

**Objective:**

Primarily, this study was aimed at investigating menstrual hygiene management (MHM) practice and determinant factors among young adolescent school girls in eastern Ethiopia, Gursum District 2021.

**Method:**

An institutional-based cross-sectional survey was conducted among adolescent school girls in Gursum, Eastern Ethiopia, in 2021. 577 girls participated in this study and a multi-stage sampling procedure was employed so as to select a fair and representative sample of female students who experienced menarche. After controlling for confounding variables, binary logistic regression was fitted to identify factors affecting MHM among adolescent girls.

**Result:**

This study revealed that 58.41% of adolescent school girls practice unsafe MHM practices. It was also reported that 193(33.45%) of the girls use reusable sanitary pads. Of those, 182(31.5%) of them keep the pads in hidden places as it is a shame to be seen Seventy-six (13.17%) of the respondents had experienced vaginal infections during menarche. Having knowledge about menstruation prior to experiencing menstruation [AOR 0.28 CI: (0.1476132, 0.5613692)], being over 15 years old [AOR 1.56, CI: (1.020577, 2.387646)], living in rural areas [AOR 1.23 CI: (1.1563013, 1.3562546)], and having infection around their vagina during menarche [AOR 4.6 CI: (2.633405, 8. 273,883)] were significant determinants of MHM practice.

**Conclusion:**

The majority of the adolescent girls who participated in this study practice unsafe MHM practices. Results suggest that school health education focusing on improving the hygienic practices of adolescent girls during menstruation should be provided.

## Introduction

Even though menstruation is a natural process, it is associated with several misconceptions and malpractices which may cause serious ill-health, including reproductive tract and urinary tract infections [1]. Globally, 2.3 billion girls and women do not manage their menstruation properly due to a lack of menstrual hygiene management (MHM) facilities, high costs, and ignorance. This leads to the use of other options, such as old clothes or other unhygienic materials, which may make them a risk group for infections and other health consequences [2]. Currently, there is adequate management of menstrual hygiene in developed countries. However, it is a major problem among girls and women in poor countries, which potentially affects their health and development [3].

About 200 million women and girls from developing countries struggle to get access to clean water for personal hygiene, and private places [4]. They face major challenges as a result of poor hygiene facilities due to inadequate water supply for washing, lack of soap, lack of privacy, non-functioning or unclean toilets, and no disposal facilities for menstrual management in the school environment [3, 5–7].

Myths, taboos, and socio-cultural-related factors create barriers for adolescents in getting adequate information regarding proper menstrual management in low- and middle-income countries. These problems limit their routine activities and negatively affect their self-esteem, reproductive health, as well as schooling [8, 9].

Despite the significant role of appropriate menstrual hygiene practices, it is still a missed opportunity to address the hygienic practice of menstruation among girls in many low and middle income countries, including Ethiopia [10].

In Ethiopia, the majority of girls are at risk of getting genitourinary tract infections as a result of poor hygienic practices during their menstruation period [11]. Most girls do not communicate with their family about menstrual hygiene due to fear of the punishments and prevented from school [12]. Even, existing sanitation conditions in majority of schools in Ethiopia are inadequate that has potential impact on girls’ education [13]. According the previous study conducted in Ethiopia, about 43.0 to 54.5% of girl students were absent from school for 1 to 4 days in each menstrual period as result of menstruation-related problems [14, 15].

Besides these problems, there is limited information on menstruation and its hygienic management as well as its influence on girls’ education in Ethiopia [15]. Therefore, the current study is aimed to assess the hygienic menstrual management and associated risk factors among adolescent school girls in Gursum district, Eastern Ethiopia.

The findings of the current study will be used by policymakers, stakeholders, health program planners, researchers, and other bodies responsible for solving these problems by providing adequate information regarding menstruation and menstrual hygiene or by providing appropriate intervention programs for schoolgirls.

## Methods and material

### Study setting, and design

An institutional-based cross-sectional survey was conducted among adolescent school girls in Gursum, Eastern Ethiopia. A total of 40 schools are available in the district, five of which are found in the town and the other 35 in the rural kebeles. Gursum is distanced from Addis Ababa and Harar by 598 km and 75 km, respectively. The total population of the district is 151,931, of which 77,112 are men and 74,819 are women. The living condition of the people, both in rural and urban areas, is dependent on cash crops like Khat, ground nuts, and coffee [16].

### Participants

All female students from grade 8–12 who experienced menarche and all the schools found in the district are source population. Representative sample was drawn female students’ who experienced menstruation and were available at school during the data collection was included in the study. Female students in the same grade level who experienced menarche but were seriously sick and absent from school at the time of data collection was note excluded from the study.

### Sample size and sampling procedure

The total number of source population was 3237.Sample size was obtained using the 50% proportion of female students considered as properly practicing menstrual hygiene. 50% proportion of female students were taken because we could not find similar study conducted prior to this study in Ethiopia. A precision of 5% and with 95% confidence level was taken and by considering 10% non-response rates and design effect of 1.5 final obtained sample size was 634.Finaly, the response rate obtained was 91%.

**Fig. 1:**
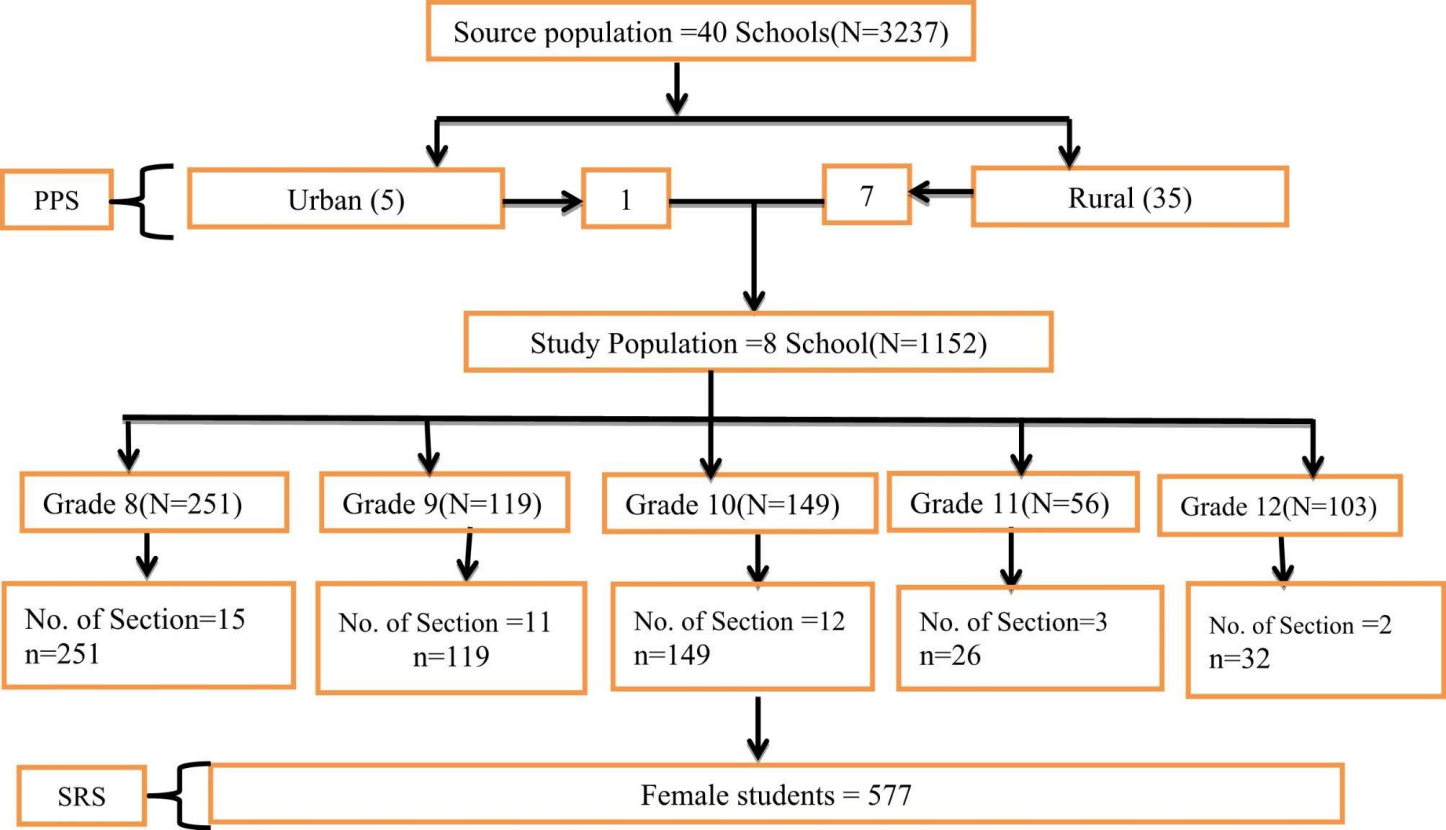
Schematic Presentation of sample size and sampling technique

A multi stage sampling procedure was employed so as to select a fair representative sample from 1152 female students who experienced menarche and attending class in the selected 8 schools. First, from a total of 40 schools 5 from urban and 35 from rural, 1 and 7 schools were selected proportionally using simple random sampling respectively. From each selected schools, sections and then individual female students who started menarche at the time of data collection was selected using simple random sampling (Fig. 1).

### Data collection tool, procedures, and quality control

A self-administered questionnaire adopted from the UNICEF WASH manual [17] and reviewing other relevant literature was used to collect quantitative data [18, 19]. Due to the sensitivity of the issue and to get reliable data without fear, four female data collectors (midwifery) and one supervisor were recruited from Gursum health centers. Intensive training was given for two days by the principal investigator on the process of data collection and critical ethical issues. In the Babille district of eastern Ethiopia, a pretest was conducted at Karl Hems Bom high school., eastern Ethiopia.

### Data processing and analysis

After checking for completeness, collected data was entered to Epi data version 3.0 and exported to Stata version 17 statistical software for descriptive and inferential analysis. Bivariable analysis was done to determine predictable variables which has a significant association with outcome variable. Predictor variable which showed a significant association with MHM practice in bivariable analysis at p-value ≤ 0.25 were fitted into multivariable analysis. Finally, independent variables were declared statistically significant at 95% confidence interval (CI), and P < 0.05.

### Outcome variable measurement (unsafe MHM practice)

World Health Organization and UNICEF’s proposed definition of menstrual hygiene management as “1.use of clean menstrual management material to absorb or collect menstrual blood that can be changed in privacy as often as necessary for the duration of a menstrual period, 2. using soap and water for washing the body as required, and 3.having access to safe and convenient facilities to dispose of used menstrual management materials (disposed in a latrine or buried) during menustruation.Accordingly, the school girls who fulfill the above three criteria during menstrual time was categorized as Safe MHM and those who do not practice at all, practice only one and/or two of the three criteria stated above were categorized as unsafe MHM practice [20, 21].

## Results

### Sociodemographic characteristics

Out of the total, 577 study participants, more than half (n = 299, 51.82%) of them were urban residents. The mean age at first menarche was 14 (SD 1.35). Out of a total 577 study participants, 125(42.65%) were from grade eight, while the rest were from grades 9–10 and 11–12 respectively. (Table 1).

**Table 1:** Socio-demographic characteristics of school adolescent girls in eastern Ethiopia, Gursum district 2021

Variable	Frequency	Percentage
**Age**		
13–15 years	174	30.16
> 15 years	403	69.84
**Residence of respondents**		
Urban	299	51.82
Rural	278	48.18
**Level of education**		
Grade 8	251	43.50
Grade 9–10	268	46.45
Grade 11–12	58	10.05
**At what age have you experienced the first menstrual cycle**		
13–15 years	206	35.70
> 15 years	371	64.30

### Knowledge about menstrual hygiene

The great majority of 520(90.12%) respondents had heard about menstruation before they experienced menarche. Female friends, 209 (36.22%), were the major source of information about the menstrual cycle. The result from self-report also indicated that 275(47.66%) of the participants have experienced an irregular menstrual cycle. Regarding the duration of menstruation, 432(92.20%) of them responded that, on average, the duration of menstruation lasts up to five days. On the other hand, 514 (89.08%) of the adolescent girls knew commercially available sanitary pads (Table 2).

**Table 2:** Knowledge of menstrual hygiene among of school adolescent girls in eastern Ethiopia, Gursum district 2021.

Variable	Frequency	Percentage
Have you ever heard of the menstruation cycle before your menstrual cycle started		
Yes	520	90.12
No	57	9.88
**What is the source of information?**		
Mother	132	22.88
Sister	159	27.56
Father/brother	39	6.76
Friend Female	209	36.22
**Is your menstrual cycle regular or irregular in a month?**		
Regular	302	52.34
Irregular	275	47.66
**For how many days does your menstrual cycle last**		
up to 5 days	532	92.20
> 5 days	45	7.80
**Do you know the commercially available sanitary pad?**		
Yes	514	89.08
No	63	10.92

### Hygienic practice during menarche

According to the data obtained from the respondents,369 (63.95%) of respondents use disposable sanitary pads, while 15 (2.60%) of the respondents use only underwear during menstruation. Of those who use disposable sanitary pads, 341 (59.10%) use commercially available sanitary pads. Further, among the respondents who use reusable sanitary pads, 170(29.46%) use pieces of cloth as an absorbent pad. Out of those who use reusable sanitary pad 182(31.54%) keep it in hidden places because it is perceived as a shame to be seen. More than half (332 (57.54%) of the respondents wash their genitals more than three times per day. It was also reported that 76 (13.17% of them) had experienced vaginal infection during menarche (Table 3).

**Table 3:** Hygienic practices during menstruation among of school adolescent girls in eastern Ethiopia, Gursum district 2021

Type of sanitary pad used		
Disposable MHM	369	63.95
Re-usable MHM	193	33.45
Underwear alone	15	2.60
**type of disposable menstrual hygiene management materials do you use**		
Sanitary pad from market	341	59.10
Toilet paper	8	1.39
Cotton	15	2.60
Sponge	5	0.87
**What type of reusable menstrual hygiene management materials do you use**		
Piece of cloth	170	29.46
Piece of sponge	15	2.60
Other	8	1.39
**Where do you keep or put reusable menstrual hygiene management materials**		
Hidden Place	182	31.54
hidden place	7	1.21
Other	4	0.69
**Why do you keep in hidden Place**		
Shame/not good to sight	163	28.25
It is not allowed	15	2.60
Other	4	0.69
**Have you experienced infection or skin rash during menstrual cycle**		
Yes	76	13.17
No	501	86.83
**How often do you wash genital during the menstrual cycle?**		
Once per day	48	8.32
Twice per day	197	34.14
More than three times per day	332	57.54
**How do you wash genitals during the menstrual cycle?**		
Only water	220	38.13
Water and soap	357	61.87
**Proper disposal of used menstrual hygiene materials**		
Yes	225	38.99
No	337	58.41
**MHM-Practice**		
Safe Practice	240	41.59
Un-safe Practice	337	58.41

### Factors associated with menstrual hygiene management practice among adolescent women in eastern ethiopia, gursum district

The prevalence of unsafe MHM was found to be 58.41% with 95% CI (0.54, 0.62).The final multivariable logistic regression model indicated that having prior information about the menstruation cycle before menstruation started decreased the odds of unsafe MHM practice among adolescents by 72% [AOR.28 CI: (0.145, 0.56)] compared to their counterpart. The likelihood of unsafe MHM practice was 1.56 [AOR 1.56, CI: (1.02, 2.39)] times more likely among > 15 years old girls, compared to girls less than 15 years old (Table 4).

**Table 4:** Factors Associated with Menstrual Hygiene Management Practice among of school adolescent girls in Eastern Ethiopia, Gursum District 2021

Variables	MHM-Practice		COR with 95% CI	AOR with 95% CI
	Safe practice	Unsafe practice		
**Age**				
13–15 years	86	88	Ref.	
> 15 years	251	152	1.68(1.38, 2.32)	1.56(1.02, 2.39)
**Residence of respondents**				
Urban	224	75	Ref.	
Rural	113	165	1. 22 (0.36, 2.32)	1.23(0.16, 1.36)
**Level of education**				
Grade 8	127	124	Ref.	
Grade 9–10	106	162	1.49(1.05, 2.11)	0.87(0.35,1.13)
Grade 11–12	8	48	1.86 (1.04, 2.01)	1.72(0.97, 1.99)
**Have you ever heard of the menstruation cycle before your menstrual cycle started**				
Yes	319	201	Ref.	
No	18	39	0.29	0.29(0.145, 0.56)
**Experienced health problems during menstruation**				
Yes	25	51	Ref.	
No	312	189	3.36(2.01, 5.61)	4.67(2.63, 8.27)
**Do you know the commercially available sanitary pad?**				
Yes	318	196	Ref.	
No	19	44	0 0.26(0.15, 0.46)	0.32(0.17, 0.60)

As the data obtained from this study being from rural areas increases the likelihood of unsafe MHM practice by 1.23 [(AOR 1.23 CI: (0.16, 1.36), compared to their counterparts. Furthermore, knowing a commercially available sanitary pad decrease the odds of unsafe MHM practice among adolescent girls by 68% [AOR 0.32 CI: (0.17, 0.60)]. The odds of unsafe MHM practice, were 4.6 times higher [AOR 4.6 CI: (2.63, 8.27)] among respondents who had experienced a health infection around their vagina during menarche (Table 4).

## Discussion

Overall, this study investigated the MHM practice and factors affecting it among young adolescent school girls in eastern Ethiopia, Gursum district in 2021. In this study, it was reported that more than half (58.41%) of the study participants practiced unsafe-MHM practices. This finding is comparable with a previous studies conducted among adolescent school girls in Ambo, Holeta, Nekemte town, and Indonesia where the prevalence of unsafe MHM practice was 53.6%, 63.3%, 60.1%, and 64.1%, respectively [22–25]. On the other hand, the findings is lower than a report from Bahirdar and Uganda, where only one in four and 9.5% of them practice safe MHM [26, 27]. Perhaps, the discrepancy may be due to differences in sociodemographic status, study time frame, and study setting.

It was also reported that unsafe MHM was more likely among those older than 15-year-old adolescent girls compared to their counterparts. However, studies in Ghana and India indicated that safe MHM management is more likely among older girls compared to younger ones. [21, 28]. This difference could due to variation in study area and living standard.

The findings of this study also showed that the odds of safe MHM were higher among female adolescent girls who had heard about the menstruation cycle before experiencing menstruation when compared to their counterparts. Likewise, knowing a commercially available menstrual pad increases the odds of safe MHM practice by 68.6% [AOR 0.32 CI: (0.173355, 0.6047782)] among adolescent girls. This is supported by the study conducted in Nekemte town where prior information about menarche is significantly associated with MHM [24]. In addition, this finding is also supported by a study done in Mehalmeda, Amhara, which reported that safe MHM was more likely among adolescent girls who got information prior to menarche onset. Furthermore, a study from Nigeria indicated that safe MHM was common among adolescents who had premenarchial training [29]. A possible reason for this is that being aware of information about MHM enables adolescent girls to engage in safe MHM.

Participant residency was also found to be a predictor of MHM practice. Thus, an adolescent who is from an urban area practice safe MHM compared to a participant from a rural area. This result is congruent with findings from Amhara province and Aurangabad, India, which indicated safe menstrual hygiene management was lower among rural girls [30, 31]. The possible reasons may be due to better socio-economic status, better use of commercially available sanitary pads, and a good perception among urban resident girls [30, 32, 33]. However, this finding is in contrast with a comparative study conducted in Bahirdar where a significant difference was not observed between the rural and urban adolescent girls [27].

During menstruation, nearly 14% (n = 76, 13.17%) of adolescent girls have experienced vaginal infection. Furthermore, the odds of safe MHM practice were much lower when compared to their counterparts. A relatively much higher magnitude of infection was reported in a study conducted in Uganda where 47.2%, 54.3%, and 60.3% of the study participants experienced vaginal discharge, skin irritation, and itching or burning in the pelvic area during menstruation [26].

cause effect relationship. In addition, due to cultural sensitivity of the study and self-report it may subjected to social desirability bias.

## Conclusion

More than half of the girls were practicing unsafe menstrual hygiene management. Evidence from the study indicated that having prior information about the menstruation cycle, residence area, knowing commercially available sanitary pads, the experience of infection around the vagina during menstruation, and age of the respondents were found to be factors affecting menstrual hygiene management practices among adolescent girls. As a result, we recommend that all respective stakeholders should work in collaboration to improving MHM practices during menarche among school adolescent girls.

### Limitations of the study

We encountered several limitations in this study that should be considered when interpreting the findings. Data regarding the income status of a family, pocket money for the students, and the educational status of a family were not collected which might have influenced the result of this study if it had been collected. In addition, due to the cross-sectional nature of the study, it is not possible to establish a cause-effect relationship.

## Data Availability

upon reasonable request to correspondent author this study’s supporting data and material is readily available.

## Abbreviations

MHM: Menstrual hygiene management
AOR: Adjusted odd ratio
COR: Crude odd ratio
CI: Confidence Interval
